# The effect of arbutin on lipid peroxidation and antioxidant capacity in the serum of cyclosporine-treated rats

**Published:** 2015

**Authors:** Fatemeh Khadir, Mahdi Pouramir, Seyyed Gholamali Joorsaraee, Farideh Feizi, Hadi Sorkhi, Fatemeh Yousefi

**Affiliations:** 1 Department of Biochemistry, Babol University of Medical Sciences, Babol, Iran.; 2 Cellular and Molecular Biology Research Center, Babol University of Medical Sciences, Babol, Iran.; 3 Fatemehzahra Infertility and Reproductive Health Research Center, Babol University of Medical Sciences, Babol, Iran.; 4 Department of Anatomy, Babol University of Medical Sciences, Babol, Iran.; 5 Non-Communicable Pediatric Diseases Research Center, Babol University of Medical Sciences, Babol, Iran.

**Keywords:** *C* yclosporin A, Oxidative stress, Arbutin, Antioxidant

## Abstract

**Background:**

Cyclosporine A (CsA) is a potent immunosuppressant drug with therapeutic and toxic actions. The use of CsA is limited by its toxicity. Several researchers had proposed that oxidative stress could play an important role in CsA-induced toxicity. Arbutin has recently been shown to possess antioxidative and free radical scavenging abilities. The present study was designed to investigate the in vivo effects of arbutin on lipid peroxidation and antioxidant capacity in the serum of cyclosporine treated rats.

**Methods:**

Adult male Wistar rats were divided into six groups (n=8/group): (I) control (no CsA and arbutin administration), (II and III) were treated subcutaneously (Sc) with arbutin (50,100 mg/kg/bw), respectively, (IV) administered CsA (25 mg/kg/bw) intraperitoneally (IP), (V and VI) received the combination of CsA (25 mg/kg/bw) i.p and arbutin (50,100 mg/kg/bw) Sc daily, respectively. At the end of the treatment (after3 weeks), serum lipid peroxidation was measured by thiobarbituric acid-reacting substances (TBARS) and serum total antioxidant capacity (ferric reducing ability of plasma [FRAP]) was assayed based on spectrophotometric method.

**Results:**

TBARS had been significantly increased by CsA administration compared with control rats. Arbutin (50mg/kg/bw) completely prevented this effect, but arbutin (100 mg/kg/bw) alone or in combination with CsA significantly increased lipid peroxidation compared with controls.

**Conclusion:**

Our data indicate that arbutin (50mg/kg/bw) had protective effect in the CsA-induced toxicity but high concentration of arbutin (100mg/kg/bw) showed meaningful oxidative and lipoperoxidative effects.

**C**yclosporine A (CsA) is an effective and powerful immunosuppressant drug universally used for the prevention of allograft rejection in solid organ transplantation and for the treatment of autoimmune diseases. However, CsA therapy is accompanied with the frequent and dose dependent side effects such as nephrotoxicity, hypertension, gingival hypertrophy and hepatotoxicity. Nephrotoxicity is the most important complication of the therapy with CsA (1-3). It has been shown that the negative effects of CsA cause histopathological changes in various organs, i.e. thymus, kidney, liver, heart, pancreas and nervous system (3). Vascular damage is a common factor in all types of CyA-induced organ injury (4, 5). 

CsA administration increases hypertension and therefore, increases the thickness of the arteriolar walls and decreases the size of the vessel lumen leading to ischemia and glomerulosclerosis. Increased blood pressure can directly damage the glomeruli by increasing the intraglomerular hydrostatic pressure (6). The mechanisms of CsA-induced toxicity were unknown, but several researchers proposed that oxidative stress could play an important role in CsA-induced toxicity. They seem to be related with increased production of free radicals and oxidative stress. Reactive oxygen species (ROS) can be produced either directly from CsA or during its metabolism by the cytochrome P450 (Cyt P450 3A) system (7).

Much experimental evidence was reported that CsA induced imbalance between production of ROS and antioxidant defence systems. Indeed when oxidative stress overcomes on antioxidant defense, lipid peroxidation progresses (8-11). Previous studies showed that CsA increased lipid peroxidation products, TBARS, decreased glutathione in the rat kidney and liver and impaired antioxidant defence system (10).

The involvement of ROS in CsA toxicity was confirmed by the fact that many antioxidants and free radical scavengers provided marked functional and histopathological protection against CsA toxicity (1, 8). Many herbs have been demonstrated in vitro and invivo experiments to protect against oxidative damage by inhibition or quenching free radicals and reactive oxygen species. *Pyrus biossieriana bush* is one of the pear species that is native to Iran. The pear leaves contain a considerable amount of arbutin, a glycosilated hydroquinone that inhibits tyrosinase, it has also been reported to have a variety of pharmacological activities and therapeutic properties as anti-inflammatory, antiviral, antihyperglycaemic, antihyperlipidemic, antioxidant, free radical scavenging, gastroprotective and alpha-glucosidase and alpha-amylase inhibitory activities (11-14). The aim of this study was to investigate the possible beneficial effects of arbutin on CsA-induced oxidative damage.

## Methods

**Animals:** Forty-eight male Wistar rats, weighing 150–170 g, were obtained from animal facility (Babol University of Medical Sciences, Babol, Iran). The animals were kept in wire-mesh floored cages under standard laboratory conditions of 12 h/12 h light/dark, 25±2 ^o^C and given a standard rat chow diet and water ad libitum. The experimental protocols were approved by the Research Committee of Babol University of Medical Sciences. The research was conducted according to the guidelines of Institutional Animal Ethics Committee (IAEC).

**Drugs and chemicals:** CsA was purchased from (Novartis Pharma AG, Basel, Switzerland). Thiobarbituric acid (TBA) and 2, 4, 6-tripyridyl-s-triazine (TPTZ), were obtained from Sigma Aldrich Chemical Company. Arbutin was obtained from Sigma-Aldrich (Germany) and was dissolved in saline and all other chemicals and solvents were of the highest available commercial grade.

**Experimental protocol:** The rats were randomly divided into six groups of eight animals each and were treated for 21 days as follows: Group I rats served as control (no CsA and arbutin administration).Groups II and III: arbutin (50, 100 mg/kg/day) administered Sc, respectively.Group IV was treated with CsA (25 mg/kg/day) i.p .Group V and VI received the combination of CsA (25 mg/kg/bw) and arbutin (50 ,100 mg/kg/day), respectively.The dose of CsA chosen was based on previous studies which had been demonstrated to successfully and consistently induce toxicity (8).

The animals were anesthetized with chloroform and sacrificed 24 hours after the last injection. Blood samples were collected from axillary artery of rats. Serum were obtained by centrifugation used for measurements of TBARS and FRAP.

**Evaluation of serum MDA:** The thiobarbituric acid-reacting substances (TBARS) assay was used to measure serum lipid peroxidation which estimated malondialdehyde (MDA) concentration. The working TBARS solution contained 0.375% TBA and 15% TCA (trichloroacetic acid; sigma) in 0.25 N HCl; TCA-TBA-HCl reagent was freshly prepared and 2 ml of this solution was mixed with 0.5 ml serum or standard in a test tube. The solution was heated for 15 min in a boiling water bath. After cooling, the absorbance was measured at 532 nm against a blank that contains all the reagents minus serum. Different concentrations of MDA (0-9-18-30 μM) were used as the standards.

**Evaluation of total antioxidant activity:** Total antioxidant activity was determined by the FRAP (ferric reducing antioxidant power) assay (15). The principle of this method is based on the reduction of a ferric-tripyridyltriazine [Fe (III)-TPTZ] complex to the ferrous-tripyridyltriazine [Fe (II)-TPTZ] form in the presence of antioxidants. The FRAP reagent was prepared from10 mmol/L TPTZ solution in HCl 40 mmol/L plus FeCl3 (20 mmol/L) and acetate buffer (0.3 mol/L, pH 3.6) in a 1:1:10 ratio. Freshly prepared FRAP reagent was warmed at 37 ^o^C for 5 min. Serum sample or standard (50 μL) was mixed with 1.5 ml of FRAP reagent in a test tube and incubated at 37^o^C for 10 min. Then the absorbance of the colored products (ferrous tripyridyltriazine complex) was measured at 593 nm and compared to the blank. FeSO4 solutions at various concentrations (125-250-500- 1000 μM) were used as standards.

**Statistical analysis:** Differences between groups were determined by one-way analysis of variance (ANOVA) with post-hoc test (Tukey). P<0.05 was defined statistically significant.

## Results

**Fig 1:**
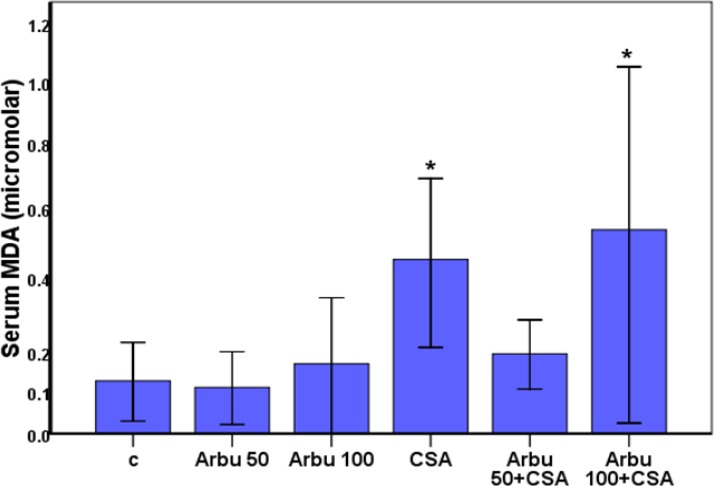
Effect of arbutin on CsA-induced lipid peroxidation in the serum of rats. Data are expressed as mean ± SEM)

Serum lipid peroxidation (TBARS) in the CsA-treated rats increased significantly (*P<*0.03) as compared to the control group. A simultaneous treatment of arbutin (50mg/kg) significantly prevented the increase in TBARS generation. Arbutin (100mg/kg) in combination with CsA significantly increased (P<0.008) the level of TBARS generation compared to the control group (figure 1).

* (P<0.05 as compared to control group. (c=control, Arbu 50=arbutin 50 mg/kg/bw, Arbu 100=arbutin 100 mg/kg/bw, CSA=CSA25 mg/kg/bw, Arbu 50+CSA= arbutin 50 mg/kg/bw+ CSA25 mg/kg/bw, Arbu100+CSA= arbutin 100 mg/kg/bw+ CSA25 mg/kg/bw).

**Fig 2:**
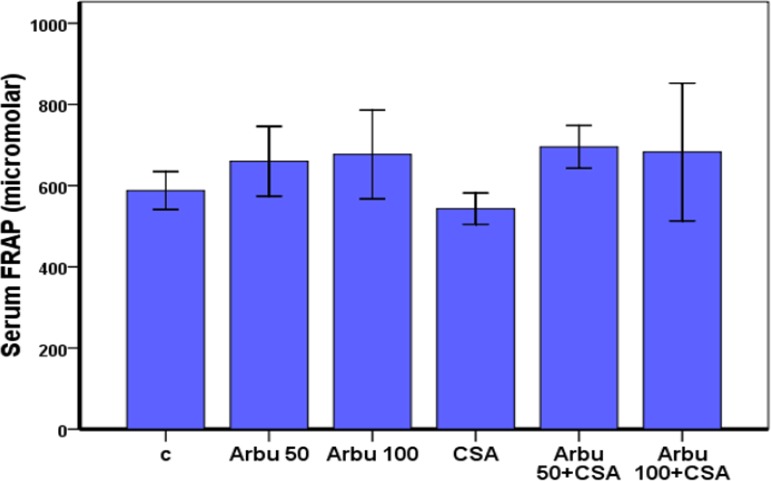
Effect of CsA and Arbutin on total antioxidant capacity in the serum of treated rats.total antioxidant power in serum was measured by FRAP assay. Values are presented as mean ± SEM

The serum antioxidant activity (FRAP) in CsA-treated rats was reduced, but there were no significant differences between the groups (figure 2).

## Discussion

There is much evidence that *suggest* oxidative stress may play an important role in CsA-induced toxicity (16). In the present study, we demonstrated that CsA administration (25 mg/kg/bw, i.p., 21 days) significantly enhances lipid peroxidation in the serum as revealed by the increased serum (TBARS). In our study, we demonstrated that arbutin has protective effects against CsA -induced toxicity. Arbutin at a high concentration in Pyrus biossieriana Buhse extract, was selected on the basis of its free-radical scavenging power and low toxicity in vivo (11, 17). Our data show that arbutin at dose 50 mg/kg/bw completely counteracts CsA-induced oxidative stress as indicated by the decreased serum lipid peroxidation.

Although the exact mechanism of *CsA-induced ROS* formation is not completely understood, numerous current findings suggest that the following factors may be involved in the *production* of *ROS:* increasing lipid peroxidation in blood and *metabolism* of CsA by cytochrome P450 oxidase (P450 3A) in the liver microsomes. According to previous studies CsA activates phospholipase A2 (PLA2) in the blood and kidney tissue and causes metabolite arachidonic acid activation (18, 19). Parra Cid (2003) showed that CsA increases the expression of cyclooxygenase (COX-2), a crucial enzyme in arachidonic acid metabolism, in glomerular cells (20) resulting in eicosanoid biosynthesis. During these reactions, free radicals, prostaglandins, leukotrienes, thromboxanes and malondialdehyde (MDA) are synthesized. CsA is able to increase the glomerular synthesis of superoxide anion, hydrogen peroxide and thromboxanes (TX) (20). 

Our study suggested that CsA or its metabolites produced free radical species that attacked lipid components, leading to lipid peroxidation. Previous studies reported that arbutin provides antioxidative effects for lipids of membrane. Studies confirmed that in addition to having indirect effects of arbutin at stabilizing membrane, it directly preserves membranes at the oxidative situation (21). Oliver (1996) showed that arbutin inhibits the enzyme phospholipase A2 in partially dehydrated liposome (22) and , the *inhibition effect of arbutin* is probably mediated by a direct effect of arbutin on the cell membrane (21, 23). 

We hypothesized that arbutin, at dose of 50 mg/kg/bw, would *inhibit* the release of arachidonic acid from the membrane via inhibition of phospholipase A2 and therefore decrease lipid peroxidation and free radical production. The present study shows arbutin 100 mg/kg/bw increases lipid peroxidation. It has also been shown that the combination of CsA and arbutin 100 mg/kg/bw increase lipid peroxidation. In fact, arbutin 100 mg/kg/bw has lipoperoxidative effect and induces oxidative damage.

In conclusion, our data indicate that arbutin 50 mg/kg/bw is able to completely prevent lipid peroxidation induced by CsA, and arbutin 100 mg/kg/bw has the oxidant and lipoperoxidative effect.

## Notes

**Funding: **This paper is the result of medical student thesis and has been ﬁnancially supported by Shahed University, Tehran, Iran.
